# Non-Invasive Blood Cortisol Estimation from Sweat Analysis by Kinetic Modeling of Cortisol Transport Dynamics

**DOI:** 10.3390/s25154551

**Published:** 2025-07-23

**Authors:** Xiaoyu Yin, Sophie Adelaars, Elisabetta Peri, Eduard Pelssers, Jaap den Toonder, Arthur Bouwman, Daan van de Kerkhof, Massimo Mischi

**Affiliations:** 1 Department of Electrical Engineering, Eindhoven University of Technology, 5612 AZ Eindhoven, The Netherlands; 2 Catharina Hospital, 5623 EJ Eindhoven, The Netherlands; 3 Department of Mechanical Engineering, Eindhoven University of Technology, 5612 AZ Eindhoven, The Netherlands

**Keywords:** patient monitoring, kinetic modeling, inverse modeling, cortisol

## Abstract

We present a novel method to estimate blood cortisol concentration from sweat cortisol measurements, incorporating a kinetic model to simulate cortisol transport dynamics. Cortisol dysregulation is observed in conditions like Cushing’s syndrome, characterized by excessive cortisol production, and stress-related disorders, which can lead to metabolic disturbances, anxiety, and impaired overall health. Sweat-sensing technology offers a non-invasive and continuous alternative to blood sampling. However, the limited research exploring the sweat–blood cortisol relationship in patients shows a moderate correlation (R<0.6), hindering its clinical application for long-term monitoring. In this paper, we propose a novel kinetic model describing cortisol transport from blood to sweat. The model was validated using data from 44 patients before and after cardiac surgery. A high Pearson correlation coefficient of 0.95 (95% CI: 0.92–0.97) was observed between our model’s estimated and experimental blood cortisol concentrations. Moreover, the method enables personalized estimation of physiological parameters, accurately reflecting patients’ status under varying clinical conditions. The method paves the way for the clinical application of long-term, non-invasive monitoring of cortisol using sweat-sensing technology. Enabling the personalized estimation of physiological parameters could potentially support clinical decision-making, helping doctors diagnose and monitor patients with health conditions involving cortisol dysregulation.

## 1. Introduction

Cortisol is a steroid hormone that plays a critical role in the body’s response to several health-related conditions. Blood cortisol concentrations are widely used in clinical practice to diagnose conditions such as Cushing’s syndrome [1,2,3], characterized by excessive cortisol production, and Addison’s disease [4,5], marked by insufficient cortisol concentrations. Both conditions can severely affect metabolism, blood pressure, and overall health. Beyond diagnosis, some studies have also hypothesized a link between elevated blood cortisol concentrations and an increased risk of postoperative delirium [6,7]. Furthermore, the prolonged monitoring of blood cortisol is essential for stress-related syndromes such as major depressive disorder, as elevated blood cortisol concentrations are closely linked to its progression [8]. Recent research highlights the importance of developing methods for the continuous monitoring of blood cortisol concentrations to optimize treatment and prevent recurrence in the aforementioned diseases [9,10]. However, traditional blood sampling is invasive [11], causes discomfort, and often requires frequent hospital visits, impacting patients’ quality of life. These limitations make it unsuitable for prolonged monitoring and underscore the urgent need for non-invasive techniques that can support long-term clinical monitoring of blood cortisol. Such advancements could facilitate remote patient care by healthcare professionals and ultimately improve patients’ well-being.

Sweat, as a non-invasive body fluid, holds potential as an alternative to traditional invasive blood sampling for prolonged cortisol measurement [12]. Cortisol measurements in saliva and urine have been suggested as preliminary diagnostic indicators of conditions like Cushing’s syndrome [1,3]. However, their use is typically limited to discrete sampling events, which may pose challenges for continuous or long-term monitoring. In addition, while saliva- and urine-based diagnostic methods can provide valuable diagnostic information, they may have limitations in detecting complex cases, particularly in identify the underlying causes of Cushing’s syndrome in complex cases [1,2]. Regarding depression, blood cortisol has been shown to provide more reliable and robust diagnostic insights compared to saliva or urine [13].

In contrast, sweat can be collected continuously using non-invasive sweat-sensing technology, making it a promising medium for long-term monitoring in clinical settings [12,14,15]. Some studies have investigated sweat cortisol concentrations for diagnostic and monitoring purposes [9,10,16]. However, only a limited number of studies have explored the correlation between blood and sweat cortisol concentrations. Torrente-Rodríguez et al. [17] proposed a linear relationship between blood and sweat cortisol and used linear regression to estimate blood concentrations from sweat. Their results, based on a small dataset including 8 healthy individuals, reported a correlation coefficient of 0.87. A recent study from our group investigated the blood–sweat cortisol relationship in patients before and after cardiac surgery [18], reporting a moderate correlation of 0.51 based on data from 50 patients. While previous analyses relied on simple linear regression [17,18], the complexity of the physiological mechanisms involved might not be fully captured by such an approach. Therefore, advanced modeling that accounts for the physiological transport mechanisms of cortisol in the human body may have the potential to provide a more accurate relationship between blood and sweat cortisol, thereby advancing the clinical application of sweat cortisol monitoring.

In a previous study by our group, we proposed an innovative model-based strategy for glucose estimation, which successfully estimated blood glucose based on sweat concentrations [19]. However, the transport mechanisms of glucose and cortisol differ significantly. Glucose transport is dominated by convection and diffusion between blood capillaries and the sweat gland duct. In contrast, cortisol transport involves more complex mechanisms. In blood, approximately 80–90% of cortisol is bound to corticosteroid-binding globulin (CBG), and around 5–10% binds to albumin, leaving only about 5% as free cortisol available for transport [20,21]. Additionally, evidence suggests that the enzyme 11-β-hydroxysteroid dehydrogenase type-2 (11β-HSD2), present in the sweat gland duct, converts cortisol into cortisone [22,23], further reducing cortisol concentrations in sweat. It should be noted that the values of the physiological parameters reported in the literature, such as the ratio of free cortisol to total blood cortisol, are average values derived from large populations, which may not accurately capture individual variability [21,24]. Therefore, personalizing model parameters to achieve patient-specific estimations is crucial for improving the model’s applicability and accuracy.

The aim of this paper is to propose a novel method for estimating blood cortisol concentration from sweat cortisol concentrations by integrating cortisol transport dynamics into kinetic modeling. This approach provides both accurate estimates of blood cortisol and personalized estimation of physiological parameters. The method was validated using clinical data from cardiac surgery patients, whose cortisol concentrations exhibited substantial variation before and after surgery, demonstrating its applicability to various cortisol concentrations.

## 2. Methods

### 2.1. Clinical Trial Dataset

This study was conducted using cortisol data from an observational clinical trial (ClinicalTrials.gov, ID: NCT05209555), which received approval from the local ethical review board and the Medical Research Ethics Committees United. Written informed consent was obtained from all participants, and the study adhered to the Declaration of Helsinki (Fortaleza, Brazil, October 2013) and Dutch legal regulations. The trial included 50 patients scheduled for aortic valve replacement (AVR) surgery, with sweat and blood samples collected before and after surgery, as reported by Adelaars [18], including details on the sample protocol. Among the 50 participants, 82% were male, with a median age of 73 years (IQR: 7). Thirty-four patients received 1 mg/kg dexamethasone during surgery. Six patients were excluded due to insufficient sweat volume before and/or after surgery, which prevented accurate cortisol quantification. The final analysis included 44 patients with paired sweat and blood cortisol concentrations and sweat rate measurements collected pre- and post-surgery.

### 2.2. Cortisol Transport Model

**Figure 1:**
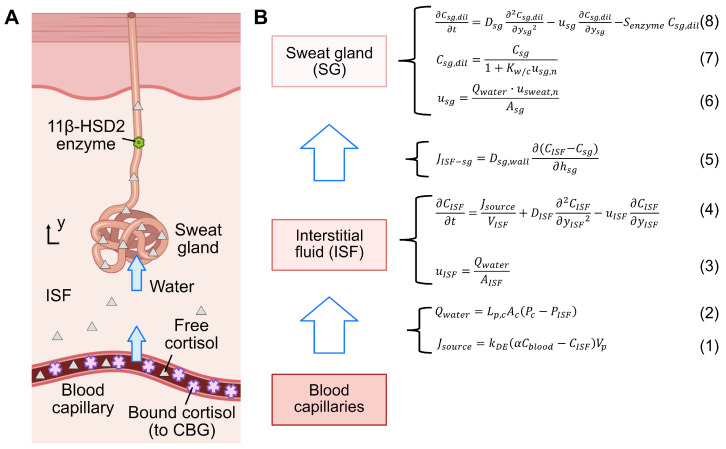
(**A**) Illustration of a proposed pathway for physiological cortisol transport from blood capillaries, through the interstitial fluid (ISF), to the sweat gland. (**B**) Schematic representation of the corresponding cortisol transport model, including compartments and associated equations. Details on the equations can be found in Section 2.2.

We propose a kinetic model for cortisol transport to characterize the mechanism of cortisol transport from blood to sweat, building upon our previous work on glucose and urea [19,25]. Unlike the glucose model, the cortisol model incorporates specific transport dynamics, including the free cortisol ratio in blood and the enzymatic activity related to cortisol-cortisone conversion, and simplifying the water dynamics to enhance model robustness. The model was developed using COMSOL Multiphysics^®^ 6.1 (COMSOL AB, Stockholm, Sweden) Figure 1 illustrates the overall cortisol transport model, represented as a compartmental system that captures cortisol transport through blood capillaries, interstitial fluid (ISF), and sweat glands.

In the blood capillary compartment, blood cortisol exists in two forms: free cortisol, which is unbound, and protein-bound cortisol, primarily bound to corticosteroid-binding globulin (CBG). Since free cortisol can passively be transported to the ISF [14,23], its flow rate ($J_{source}$ in [mol s^−1^]) from the blood capillary to the ISF is determined by the concentration gradient between these two compartments, and is given as

(1) $$J_{source}=k_{DE}\left(\alpha C_{blood}-C_{ISF}\right)V_{p},$$

where $k_{DE}$ in [s^−1^] is a generalized dermal clearance constant for steroids, encompassing cortisol [26], α denotes the proportion of free cortisol relative to the total blood cortisol [20], $C_{blood}$ in [mol m^−3^] represents the total blood cortisol concentration, $C_{ISF}$ in [mol m^−3^] is the cortisol concentration in the ISF compartment, and $V_{p}$ in [m^3^] denotes the volume of the blood capillary compartment [27].

The hydrostatic pressure difference between the blood capillary and ISF drives water movement from the blood capillary into the ISF. This process is governed by the Starling principle and can be formulated as

(2) $$Q_{water}=L_{p,c}A_{c}\left(P_{c}-P_{ISF}\right),$$

where $Q_{water}$ in [m^3^ s^−1^] denotes the rate of water flow, $L_{p,c}$ in [m s^−1^ mmHg^−1^] is the hydraulic conductivity [28], $A_{c}$ in [m^2^] represents the surface area for the blood capillary compartment [27], $P_{c}$ in [mmHg] is the capillary hydrostatic pressure [29], and $P_{ISF}$ in [mmHg] is the interstitial hydrostatic pressure [29]. The velocity of water flow $u_{ISF}$ in [m s^−1^] from the blood capillaries to the ISF is computed as $Q_{water}$ divided by the cross-sectional area of the ISF compartment $A_{ISF}$:

(3) $$u_{ISF}=\frac{Q_{water}}{A_{ISF}}.$$

In the ISF compartment, the transport of free cortisol is governed by diffusion and convection, and is given as

(4) $$\frac{\partial C_{ISF}}{\partial t}=\frac{J_{source}}{V_{ISF}}+D_{ISF}\frac{\partial^{2}C_{ISF}}{\partial y_{ISF}^{2}}-u_{ISF}\frac{\partial C_{ISF}}{\partial y_{ISF}},$$

where $V_{ISF}$ in [m^3^] denotes the ISF compartment volume [27], $D_{ISF}$ in [m^2^ s^−1^] represents the diffusion coefficient of cortisol in the ISF [30], and $y_{ISF}$ in [m] indicates the transport coordinate of cortisol in the ISF.

Next, the concentration gradient drives cortisol transport from the ISF into the sweat gland, which is governed by Fick’s first law and can be formalized as

(5) $$J_{ISF-sg}=D_{sg,wall}\frac{\partial (C_{ISF}-C_{sg})}{\partial h_{sg}},$$

where $J_{ISF-sg}$ in [mol s^−1^] denotes the cortisol flux from the ISF to the sweat gland compartment, $D_{sg,wall}$ in [m^2^ s^−1^] is the diffusion coefficient of cortisol across the sweat gland wall [30], $C_{sg}$ in [mol m^−3^] represents the cortisol concentration in the sweat gland, and $h_{sg}$ in [m] represents the sweat gland wall thickness [14]. Water also enters the sweat gland due to the pressure gradient, with its velocity described as

(6) $$u_{sg}=\frac{Q_{water,sg}\cdot u_{sweat,n}}{A_{sg}},$$

where $u_{sg}$ in [m s^−1^] denotes the water velocity in the sweat gland, $A_{sg}$ in [m^2^] represents the luminal area of the sweat gland [31], and $u_{sweat,n}$ is the experimental sweat velocity normalized by the passive sweat velocity [32]. The term $u_{sweat,n}$ serves as a correction factor for adjusting $u_{sg}$, addressing the differences between passive sweating and stimulated sweating observed under experimental conditions.

Subsequently, cortisol is transported along with water flow into the sweat gland, where it undergoes dilution upon entry. The cortisol concentration $C_{sg,dil}$ in [mol m^−3^] after dilution can be described as

(7) $$C_{sg,dil}=\frac{C_{sg}}{1+K_{w/c}u_{sg,n}},$$

where $K_{w/c}$ denotes the dimensionless ratio of the volume flow rate of water to that of cortisol [33], and $u_{sg,n}$ represents the sweat velocity in the sweat gland ($u_{sg}$) normalized by the velocity of passive sweating [32].

The diluted cortisol undergoes diffusion and convection within the sweat gland and is transported to the skin surface. This process is governed by

(8) $$\frac{\partial C_{sg,dil}}{\partial t}=D_{sg}\frac{\partial^{2}C_{sg,dil}}{\partial y_{sg}^{2}}-u_{sg}\frac{\partial C_{sg,dil}}{\partial y_{sg}}-S_{enzyme}C_{sg,dil},$$

where $D_{sg}$ in [m^2^ s^−1^] is the diffusion coefficient of cortisol in sweat [30], $y_{sg}$ in [m] represents the transport coordinate of cortisol in the sweat gland, and $S_{enzyme}$ in [s^−1^] denotes the turnover rate of the 11β-HSD2 enzyme in the sweat gland, which converts cortisol into cortisone [22,23]. The latter process acts as a sink, leading to a reduction in cortisol concentration in the sweat gland $C_{sg}$ in [mol m^−3^].

To assess the sensitivity of the model to parameter variations, we conducted a sensitivity analysis by evaluating how changes in model parameters influence the simulated sweat cortisol concentrations. For model parameters except the turnover rate of the 11β-HSD2 enzyme ($S_{enzyme}$), 100 samples were drawn from a uniform distribution within a ±20% range around the average parameter value from the literature (see Table 1). As the values for $S_{enzyme}$ are not available from the literature, the mean value was set as the average of its estimations. The model was run for each updated parameter value, and the corresponding simulated sweat cortisol concentrations were recorded. The coefficient of variation (CV) of these concentrations was then calculated.

### 2.3. Strategies for Blood Cortisol Estimation

**Figure 2:**
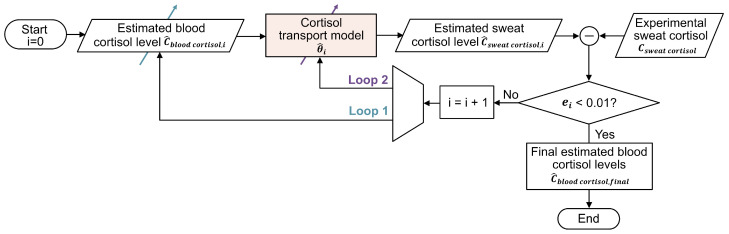
Flowchart of the double-loop optimization strategy for estimating blood cortisol concentration.

The estimation of blood cortisol concentration from measured sweat cortisol concentration was in a personalized manner by adapting a double-loop optimization strategy, originally proposed in our previous study [19]. Figure 2 illustrates the flowchart of the proposed strategy, which consists of two interleaving optimization loops. The first loop focuses on optimizing the estimated concentration of blood cortisol, while the second loop refines the physiological parameters of the cortisol transport model (see Table 1 for details), specifically targeting parameters with coefficients of variation (CV) exceeding 0.1% in the sensitivity analysis.

The approach begins by initializing the blood cortisol concentration to 355 nmol L^−1^, derived by computing the pre-surgery average of blood cortisol concentrations of our experimental data. For each iteration *i*, the optimization strategy produces an updated estimate of sweat cortisol concentration (${\hat{C}}_{\mathrm{sweat}\;\mathrm{cortisol},i}$), which is then compared with the experimental sweat cortisol concentration ($C_{\mathrm{sweat}\;\mathrm{cortisol}}$) to calculate the error ($e_{i}$) as

(9) $$e_{i}={\left(C_{\mathrm{sweat}\;\mathrm{cortisol}}-{\hat{C}}_{\mathrm{sweat}\;\mathrm{cortisol},i}\right)}^{2}.$$

The process iterates until the error meets the stopping criterion of 0.01 nmol^2^ L^−2^, at which point the final estimated blood cortisol concentration (${\hat{C}}_{\mathrm{blood}\;\mathrm{cortisol},final}$) is determined. For a detailed description of the double-loop strategy, please refer to our previous work [19].

To evaluate the estimation performance of the proposed method, we calculated the root mean square error (RMSE) between the estimated and experimental blood cortisol concentrations, along with the Pearson correlation coefficient. The evaluation was conducted for the entire dataset as well as separately for three conditions: pre-surgery, post-surgery with dexamethasone, and post-surgery without dexamethasone. A Bland–Altman analysis was performed to evaluate the agreement between experimental and estimated cortisol concentrations in blood. Additionally, the estimated proportion of free cortisol relative to total blood cortisol (α) was analyzed across these conditions using our double-loop optimization strategy.

## 3. Results

**Figure 3:**
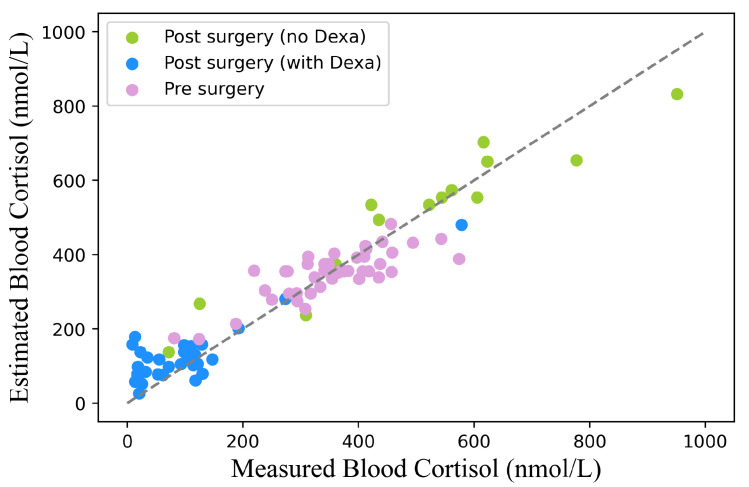
Relationship between estimated and experimental blood cortisol concentrations across three clinical conditions: pre-surgery, post-surgery without dexamethasone (Dexa) treatment, and post-surgery with dexamethasone treatment.

**Figure 4:**
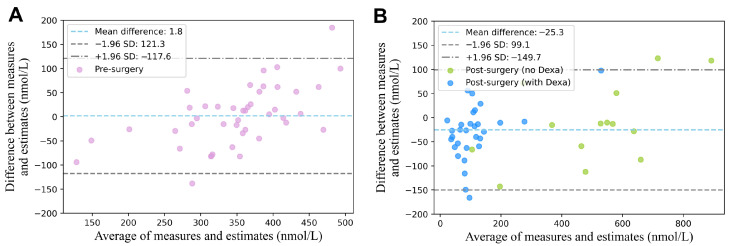
Bland–Altman analysis of estimated versus measured blood cortisol concentrations. (**A**) Pre-surgery group. (**B**) Post-surgery groups, including patients with and without dexamethasone (Dexa) treatment. Dashed lines indicate the mean difference and 95% limits of agreement, calculated as the mean difference ±1.96 times the standard deviation (SD).

Figure 3 illustrates the relationship between the estimated blood cortisol concentrations obtained using our proposed method and the experimental blood cortisol concentrations under three conditions: pre-surgery, post-surgery with dexamethasone, and post-surgery without dexamethasone. For the entire dataset, the estimation yielded an RMSE of 65 nmol L^−1^ and a Pearson correlation coefficient of 0.95 (95% CI: 0.92–0.97). When evaluated separately, the pre-surgery condition achieved a correlation coefficient of 0.79 (95% CI: 0.65–0.88) and an RMSE of 61 nmol L^−1^. For the post-surgery condition, the results were 0.87 (95% CI: 0.74–0.94) and an RMSE of 63 nmol L^−1^ with dexamethasone, and 0.95 (95% CI: 0.84–0.98) and an RMSE of 79 nmol L^−1^ without dexamethasone. The results of the Bland–Altman analysis are presented in Figure 4, evaluating the agreement between the estimated and experimentally measured blood cortisol concentrations for both pre-surgery (Figure 4A) and post-surgery (Figure 4B) conditions. The mean bias was 1.84±119.42 nmol L^−1^ for the pre-surgery data and −25.30±124.44 nmol L^−1^ for the post-surgery data.

**Figure 5:**
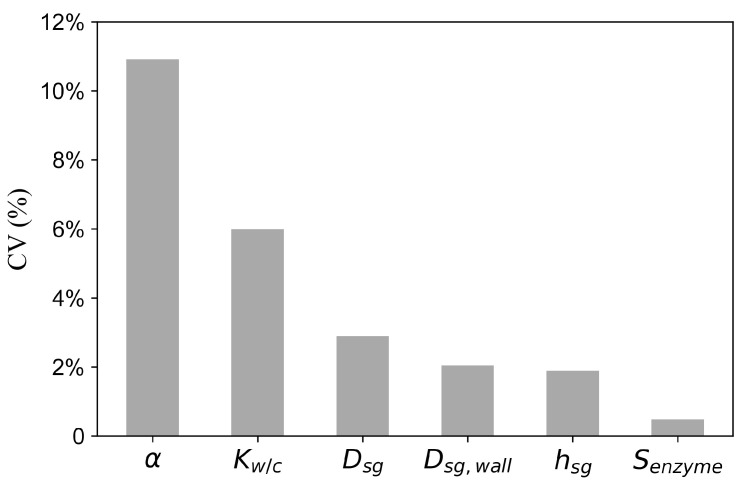
Sensitivity analysis results for parameters of the cortisol transport model, expressed in terms of the coefficient of variation (CV) of the simulated sweat cortisol concentrations. Parameters include the proportion of free cortisol (α), the ratio of volumetric flow rate of water to cortisol ($K_{w/c}$), the diffusion coefficient of cortisol in sweat ($D_{sg}$), the diffusion coefficient across the sweat gland wall ($D_{sg,\mathrm{wall}}$), the thickness of the sweat gland wall ($h_{sg}$), and the turnover rate of the 11β-HSD2 enzyme ($S_{\mathrm{enzyme}}$).

Figure 5 presents the results of the sensitivity analysis for the parameters of the cortisol transport model, showing only parameters with CV greater than 0.1%. Among all parameters, the proportion of free cortisol relative to total blood cortisol (α) had the greatest impact on the model output, with a CV of 10.9%.

**Figure 6:**
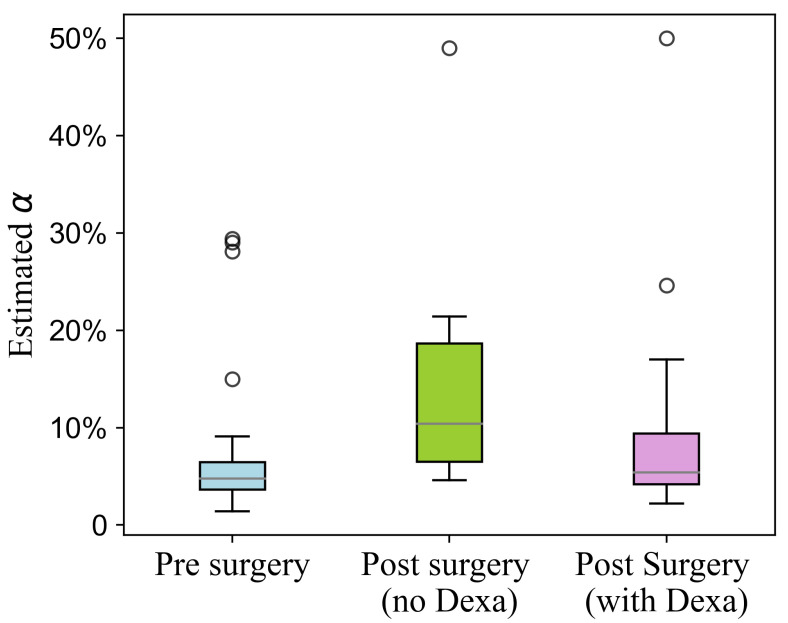
Estimated proportion of free cortisol relative to total blood cortisol (α) under three clinical conditions: pre-surgery, post-surgery without dexamethasone (Dexa) treatment, and post-surgery with dexamethasone treatment.

Figure 6 shows the estimated proportion of free cortisol relative to total blood cortisol (α), obtained using our double-loop optimization strategy. The median estimated α values were 4.8% for the pre-surgery condition, 11.3% for the post-surgery condition without dexamethasone, and 5.4% for the post-surgery condition with dexamethasone. These results highlight the variation in free cortisol proportions across different surgical and treatment conditions.

## 4. Discussion

In this paper we proposed a cortisol transport model based on the physiological transport mechanism of cortisol. This model was used with a double-loop optimization strategy to enable accurate blood cortisol estimation and personalized calibration of highly sensitive parameters in the model. These contributions collectively led to a high Pearson correlation coefficient of 0.95, indicating strong agreement between estimated and experimental values.

A previous study from our group on the same dataset reported a moderate correlation of 0.51 between the cortisol concentrations in blood and sweat in patients undergoing cardiac surgery when using linear regression [18]. Our proposed model, using the same dataset, achieved a superior correlation coefficient of 0.95, markedly outperforming the previous linear regression method. Beyond the work of [18], the only other study exploring the sweat–blood cortisol relationship is that of Torrente-Rodríguez et al. [17], which focused on healthy individuals. This study, based on a small dataset of eight subjects with four data points each, reported a correlation coefficient of 0.87. However, their analysis, which relied on a linear regression method without incorporating the physiological mechanisms of cortisol transport, yielded a lower correlation compared to our proposed model (0.87 vs. 0.95). Additionally, their dataset was significantly smaller than ours, which included 44 subjects with multiple measurements per subject.

Sensitivity analysis identified the proportion of free cortisol relative to total blood cortisol (α) as having the greatest impact on the model output, highlighting its critical role in both the cortisol transport mechanism and estimation performance. Consistent with this finding, our model parameter estimates revealed notable variations in α across different surgical and treatment conditions (Figure 5).

The observed differences in α before and after surgery align closely with the previously reported physiological mechanisms. For pre-surgery patients, the estimated free cortisol proportion (α) was 4.8%, closely matching the typical value of approximately 5% reported in the literature [20,21]. Post-surgery, the estimated α values increased in both the dexamethasone and non-dexamethasone groups relative to the pre-surgery value. Specifically, for patients not treated with dexamethasone, the estimated α was 11.3%. This increase is likely due to surgical stress, which is known for elevating cortisol production. As reported in the literature, surgical stress also leads to a reduction in CBG [24,34,35,36], the primary protein responsible for binding cortisol in blood. Lower CBG levels result in a decrease in cortisol binding, leading to a higher proportion of free cortisol. This is consistent with studies reporting a two- to four-fold increase in free cortisol after surgery. For example, Christ-Crain et al. [37] observed a 130% increase in the free cortisol index (total blood cortisol/CBG) and a 400% rise in free cortisol post-surgery. Similarly, Vogeser et al. [34] reported a 200% increase in free cortisol after surgery, while Khoo et al. [35] and Le Roux et al. [24] documented a 30% reduction in CBG levels post-surgery.

Additionally, our results demonstrated that post-surgery patients treated with dexamethasone had lower α values (5.3%) compared to those not treated with dexamethasone (11.3%). This also aligns with the literature indicating that dexamethasone strongly suppresses free cortisol concentrations compared to its effect on total cortisol in blood, potentially due to changes in the CBG saturation under dexamethasone treatment [38]. These findings support the ability of our model to accurately capture physiological variations in cortisol dynamics under different surgical and treatment conditions.

Our proposed method holds significant clinical relevance. First, we have demonstrated that blood cortisol concentrations, an essential clinical indicator for several health conditions (e.g., Cushing’s syndrome, Addison’s disease, depressive disorder, and postoperative delirium), can be accurately estimated using sweat sensing technology. This is an important achievement as the relationship between cortisol concentrations in blood and sweat had not been established yet. By modeling this relationship, our non-invasive strategy holds the potential to enable prolonged monitoring of patients undergoing cardiac surgery, reducing the need for frequent blood draws. Second, besides estimating total blood cortisol concentrations, our method can provide blood free cortisol concentrations based on the estimated proportion of free cortisol (α), which is a parameter not typically measured in standard blood tests. Since blood free cortisol concentrations are closely related to patients’ medication status and the severity of surgery [35], access to this information can assist clinicians in making more accurate diagnoses and tailoring treatments accordingly.

The proposed model builds on a framework that includes the most relevant physiological compartments involved in biomarker transport from blood to sweat. Within this framework, which was already proven valuable for explaining the transport of glucose and urea from blood to sweat [19,25], the biomarker-specific mathematical formulations of transport mechanisms are adjusted to reflect the specific physiological characteristics of each biomarker. This allows the adopted framework to flexibly accommodate various biomarkers while maintaining physiological relevance.

Despite the relevance of our findings, the generalizability of our results is limited by the relatively small sample size and the absence of a broader patient population. This study is limited by a demographically homogeneous cohort, which was predominantly male (82%) with a relatively narrow age distribution (median: 73 years, IQR: 7). Future studies should aim to recruit a more diverse cohort with balanced sex distribution and a broader age range to evaluate the generalizability of the proposed model. The data set for this study consists of 44 patients who underwent AVR surgery and does not include healthy individuals or patients with other medical conditions, such as Cushing’s syndrome. Therefore, further validation in diverse populations is necessary to evidence the model’s generalizability. In future studies with larger data sets, comparing patients across varying degrees of surgical severity could help clarify the relationship between the proportion of free cortisol (α) and the patient’s overall clinical condition. This understanding would further enhance the clinical relevance of the estimates provided by our method.

## 5. Conclusions

We have developed a novel method to estimate blood cortisol based on non-invasive sweat measurements by integrating a kinetic model of cortisol transport. This approach enables personalized estimation of both blood cortisol and physiological parameters from sweat, providing an accurate representation of patients’ physiological states under diverse clinical conditions. Our method holds notable clinical potential, providing a practical tool for long-term, non-invasive monitoring of blood cortisol via sweat-sensing technology. Additionally, the personalized estimation of physiological parameters supports clinical decision-making, aiding doctors in diagnosing and managing patients across various health situations.

## Figures and Tables

**Table 1:** Initial parameters of the cortisol transport model.

Parameter	Unit	Value	Ref.
Capillary hydrostatic pressure: $P_{c}$	mmHg	30	[29]
Capillary hydraulic conductivity of water: $L_{p,c}$	m s^−1^ mmHg^−1^	$6.5\times 10^{-10}$	[28]
Clearance constant of steroids: $k_{DE}$	s^−1^	$1.01\times 10^{-3}$	[26]
Diffusion coefficient of cortisol for sweat gland wall: $D_{sg,wall}$	m^2^ s^−1^	$2.84\times 10^{-10}$	[30]
Diffusion coefficient of cortisol in ISF: $D_{ISF}$	m^2^ s^−1^	$2.84\times 10^{-10}$	[30]
Diffusion coefficient of cortisol in sweat: $D_{sg}$	m^2^ s^−1^	$2.84\times 10^{-10}$	[30]
Effective area of sweat gland: $A_{sg}$	m^2^	$1.96\times 10^{-11}$	[31]
Effective surface area of capillary: $A_{c}$	m^2^	$1.5\times 10^{-8}$	[27]
Effective cross-sectional area of ISF: $A_{ISF}$	m^2^	$2.2\times 10^{-8}$	[27]
Effective volume of capillary: $V_{p}$	m^3^	$3.02\times 10^{-13}$	[27]
Effective volume of ISF: $V_{ISF}$	m^3^	$6.0\times 10^{-13}$	[27]
Interstitial hydrostatic pressure: $P_{ISF}$	mmHg	−3	[29]
Proportion of free cortisol: α	-	5%	[20]
Ratio of volumetric flow rate of water to cortisol: $K_{w/c}$	-	20	[33]
Thickness of sweat gland wall: $h_{sg}$	m	$5\times 10^{-5}$	[14]

## Data Availability

Data will be made available upon reasonable request.
